# Brain atrophy but not white matter lesions associate with ECT-related confusion

**DOI:** 10.1192/j.eurpsy.2024.290

**Published:** 2024-08-27

**Authors:** A. A. Politis, N. Kokras, G. Velonakis, A. M. Politis

**Affiliations:** ^1^First Department of Psychiatry; ^2^Research Unit of Radiology and Medical Imaging - Second Department of Radiology, National and Kapodistrian University of Athens, Medical School, Athens, Greece

## Abstract

**Introduction:**

Patients undergoing electroconvulsive treatment (ECT) may display an acute confusional state, often characterized by transient disorientation, inattention, memory and cognitive deficits.

**Objectives:**

In this retrospective medical chart naturalistic study, we sought the determine whether white mater lesions and brain atrophy associate with the emergence of confusion during ECT treatment and preliminary results are presented herein

**Methods:**

Medical charts of 24 consecutive inpatients with depression admitted to a psychogeriatric ward and subjected to bilateral frontotemporal ECT were examined retrospectively for patient and clinical characteristics. Mini-Mental State Examination (MMSE) and Geriatric Depression Scale (GDS) scores at admission and hospital discharge were retrospectively collected. Available brain Magnetic Resonance Imaging (MRI) scans were graded for lesions (white matter hyperintensities, WMH), parietal, temporal and global brain atrophy

**Results:**

In this pilot study of mostly elderly patients, 50% displayed signs of confusion. All patients improved substantially, as indicated by MMSE and GDS scores, irrespectively of whether they experienced transient confusion during ECT. Preliminary results indicate that WMH are unrelated to the emergence of confusion. Instead, brain atrophy, and in particular temporal lobe and mostly frontal lobe atrophy associated with confusion

**Conclusions:**

In our sample of elderly inpatients with depression subjected to bilateral ECT, preliminary results of this pilot study indicate that brain atrophy, as evidenced by MRI scans, appears as a predictor of post-ECT confusion. Moreover, the Pasquier scale, and specifically the scale sub-scores regarding brain atrophy in the frontal and temporal sulci, could prove useful in helping the clinician estimate the probability of ECT-related confusion during ECT treatment

**Disclosure of Interest:**

None Declared

